# Shift from primary pneumonic to secondary septicemic plague by decreasing the volume of intranasal challenge with *Yersinia pestis* in the murine model

**DOI:** 10.1371/journal.pone.0217440

**Published:** 2019-05-23

**Authors:** Rachel M. Olson, Deborah M. Anderson

**Affiliations:** 1 Department of Veterinary Pathobiology, University of Missouri, Columbia, MO, United States of America; 2 Laboratory for Infectious Disease Research, University of Missouri, Columbia, MO, United States of America; University of Louisville School of Medicine, UNITED STATES

## Abstract

*Yersinia pestis* is the causative agent of pneumonic plague, a disease involving uncontrolled bacterial growth and host immunopathology. Secondary septicemic plague commonly occurs as a consequence of the host inflammatory response that causes vasodilation and vascular leakage, which facilitates systemic spread of the bacteria and the colonization of secondary tissues. The mortality rates of pneumonic and septicemic plague are high even when antibiotics are administered. In this work, we show that primary pneumonic or secondary septicemic plague can be preferentially modeled in mice by varying the volume used for intranasal delivery of *Y*. *pestis*. Low volume intranasal challenge (10μL) of wild type *Y*. *pestis* resulted in a high frequency of lethal secondary septicemic plague, with a low degree of primary lung infection and rapid development of sepsis. In contrast, high volume intranasal challenge (30μL) yielded uniform early lung infection and primary disease and a significant increase in lethality. In a commonly used BSL2 model, high volume challenge with *Y*. *pestis* lacking the pigmentation locus (*pgm*-) gave 10^5^-fold greater deposition compared to low volume challenge, yet moribund mice did not develop severe lung disease and there was no detectable difference in lethality. These data indicate the primary cause of death of mice in the BSL2 model is sepsis regardless of intranasal dosing method. Overall, these findings allow for the preferential modeling of pneumonic or septicemic plague by intranasal dosing of mice with *Y*. *pestis*.

## Introduction

Historically *Yersinia pestis* has caused three pandemics, manifesting as bubonic, pneumonic and septicemic plague. Plague is usually a flea-borne disease and can occur in most mammals. Ecologically important flea-rodent transmission cycles are responsible for maintaining *Y*. *pestis* in areas throughout the world, and many years can pass between recognized human or animal outbreaks [1]. Upon deposition in the skin by flea bite, bacteria traffic to the draining lymph node and rapidly multiply. Vascular spread results in bacteremia and colonization of secondary and tertiary immune tissues as well as the lungs and liver. Secondary septicemia progresses rapidly to lethality due to disseminated vascular coagulation, acute respiratory distress syndrome, and multi-organ failure [2]. Although antibiotic treatment of bubonic plague is usually successful, the mortality rate of pneumonic and septicemic plague is high [3]. Even in countries with strong medical infrastructure, such as the US, recently reported annual mortality rates have been as high as 25%, with no age or sex bias in disease susceptibility [4]. Furthermore, reemergence of pneumonic plague has occurred in Madagascar, where in a 2017 outbreak 85% of the 2,500 cases were the pneumonic form [5]. Multi-drug resistant *Y*. *pestis* strains have been isolated from plague patients and are believed to evolve naturally in the flea-rodent enzootic cycle [6–9].

Awareness of the potential threat of bioterrorism has resulted in recent focus on *Yersinia pestis* research due to the potential for genetic engineering and person-person spread [10]. Early research focused on repurposing licensed antibiotics, but with relatively few naturally occurring human cases each year, phase III clinical trials for pneumonic plague are limited. In 2002, the US Food and Drug Administration (FDA) released guidance for licensing medical products when the disease incidence is low. The so-called Animal Rule allows for the acceptance of efficacy data from small and large animal experimental models, provided there is a strong understanding of the model and its relevance to the human response to infection and disease. In 2004, the FDA formalized pneumonic plague as a disease that falls under the Animal Rule [11]. Since this time, mouse intranasal challenge models have become widely used for the early testing of vaccines, diagnostics, and therapeutics for efficacy. Aerosol challenge models are less common in rodents, but widely used in non-human primates. Inhalation exposure systems for *Y*. *pestis* are highly specialized to manage the biosafety risks of generating highly infectious aerosolized challenge material, and very few investigators have the resources and expertise to routinely use aerosol challenge in mice. Additionally, there are cases, such as when the direct comparison of multiple bacterial strains is desired, where intranasal challenge is preferable even at facilities where aerosol challenges are feasible. There is, therefore, a pressing need to understand the mouse intranasal model so that it can be refined to better recapitulate human plague.

In addition, *Y*. *pestis* is classified as a biosafety level 3 (BSL3) tier 1 select agent with biosecurity requirements for handling. Some mutant strains of *Y*. *pestis* have been approved as lower risk and exempt from select agent requirements due to their well-characterized and irreversible attenuation. These BSL2 strains, especially the non-pigmented mutant, are commonly used for pathogenesis and immunity studies because they retain virulence in the mouse model. The pigmentation locus (*pgm*) is a 102kb chromosomal element that is spontaneously lost in laboratory culture but is present in all virulent isolates of *Y*. *pestis* [12]. Intravenous infection of mice with *pgm*- *Y*. *pestis* is lethal at a very low challenge dose, but by subcutaneous or intranasal infection, this strain is attenuated [13]. As the *pgm* locus includes a 35kb high pathogenicity island encoding yersiniabactin biosynthesis and transport for acquisition of Fe^3+^, these observations strongly suggest that growth in the lungs and infection from peripheral tissues are dependent on the yersiniabactin siderophore. However, intranasal infection of mice with high challenge doses of the *pgm*- strain leads to lethality, whereas in the bubonic model, the *pgm* mutant is avirulent [14, 15]. Mice that succumb to infection by *pgm*- *Y*. *pestis* appear to have developed systemic disease without primary lung involvement [16]. However, other laboratories have reported a higher degree of lung disease during infection with *pgm- Y*. *pestis*, and the differences for these somewhat disparate results are not well understood [17].

Pulmonary exposure to wild type (*pgm+*) *Y*. *pestis* has been suggested to proceed as a biphasic inflammatory response, with an early phase where little cytokine production and neutrophil recruitment occurs [18–20]. Vascular spread is thought to occur 36–48 hours post-infection (HPI), and allows for the infection of secondary tissues including the liver and spleen. Previous work in our laboratory and others has shown that mice challenged by respiratory infection succumb to disease 3–5 days post-infection and typically have colonization of liver and spleen when moribund [18, 21]. However, there is significant potential for variation in methods of intranasal challenge that may influence the outcome of infection. For example, it has been well-established that low volumes of therapeutics or bacteria, less than 35μL, administered to mice by intranasal instillation can result in a high degree of retention of material in the upper respiratory tract [22, 23]. In this work, we evaluated the intranasal infection technique as an experimental variable for studies on plague in the murine model.

## Materials and methods

### Bacterial strains

*Yersinia pestis* strains were routinely grown fresh from frozen stock by streaking for isolation onto heart infusion agar (HIA) plates with ampicillin (*pgm-* strain KIM5-pCD1^Ap^) or HIA plates supplemented with 0.005% (w/v) Congo Red and 0.2% (w/v) galactose (*pgm+* strain, CO92 or KIM6+pCD1^Ap^) to verify retention of the pigmentation locus [15, 24–26]. For intranasal challenge studies, a single colony was used to inoculate heart infusion broth (HIB) supplemented with 2.5mM CaCl_2_ and grown for 18–20 hours at 37°C, 125 rpm. All work with the wild type, *pgm*+ *Y*. *pestis* strains was performed in a select agent-authorized BSL3 or ABSL3 laboratory.

### Vertebrate animals

#### Ethics statement

All animal procedures were in compliance with the Office of Laboratory Animal Welfare and the National Institutes of Health Guide for the Care and Use of Laboratory Animals and were approved by the University of Missouri Animal Care and Use Committee.

Breeder C57Bl/6 mice were purchased from The Jackson Laboratory (Bar Harbor, ME). Mice used in this study were bred in-house at the University of Missouri. Mice were randomly assigned to challenge condition prior to experiments. Sample sizes and trial replicates for individual experiments are given in the figure legends. Approximately equal numbers of male and female age-matched mice, ranging from 15–30g, were used for challenge experiments. Challenge doses, prepared from the same inoculum by dilution in sterile PBS, were delivered in 10, 20, or 30μL as indicated. Mice were anesthetized in groups of 3–5 using 2.5% inhaled isoflurane with an O_2_ flow rate of 2.5 liters per minute. For intranasal instillation, 10μL doses were delivered as one droplet to the left or right naris; 20 and 30μL challenges were delivered with half of the final volume (i.e. 10 and 15μL, respectively) to each naris and the mice were not returned to the anesthesia chamber between droplets. After infection, mice were allowed to recover in a supine position with the head slightly elevated and then returned to housing. All infected mice were monitored by daily assignment of clinical score, which involved assessments of the progressive worsening of symptoms including ruffled fur, hunched posture, weakness, and reduced activity. Animals that survived to the end of the 14 day observation period or were identified as moribund (defined by pronounced neurologic signs, inactivity and severe weakness) were euthanized by CO_2_ asphyxiation followed by cervical dislocation, a method approved by the American Veterinary Medical Association Guidelines on Euthanasia.

### Infection studies

At 6, 48, or 72 hours post-infection (HPI), infected mice were euthanized and lungs (6 HPI), lungs and spleen (48 HPI), or lungs, spleen, liver, and blood (72 HPI) were collected. Trachea and other respiratory tissue were not harvested. Tissues homogenized in sterile PBS were serially diluted and plated in duplicate on HIA for bacterial enumeration. Serum was isolated following blood collection and analyzed by a multiplex cytokine assay (Millipore Sigma, Burlington, MA) for 5 pro-inflammatory cytokines known to play a role in plague: IFNγ, TNFα, IL1β, IL6, and IL10 [27, 28]. Moribund mice and those found dead were necropsied and lungs, liver, and spleens were fixed in 10% formalin and stained with hematoxylin and eosin. For quantification of pathological lesions, sample identities were blinded for analysis. Severity scoring (0–4) was based on the size and frequency of necrotic and inflammatory lesions, with 4 being the most severe. Imaging of whole lung was performed on a Lionheart FX automated microscope (Biotek, Winooski, VT) at 4X magnification in montage mode. Stitching was performed without compression in post processing.

### Liver function analysis

Mice were challenged by intranasal infection with *Y*. *pestis* KIM5-pCD1^Ap^. On day 5 post-infection, mice were euthanized, blood collected by cardiac puncture and lungs, liver, and spleen removed and homogenized in sterile PBS. Bacterial titer was determined by serial dilution. Serum was collected following centrifugation and used to measure cytokines and liver function enzymes. For cytokine analysis, serum was analyzed as above; liver function enzymes were quantified through a liver function panel by Clinical Comparative Pathology, LLC (Columbia, MO).

### Statistical evaluation

Data were generally collected in multiple independent trials as indicated in the figure legends to ensure reproducibility. Data from all trials were combined and analyzed for statistical significance. Statistical significance was evaluated using Prism 7 (GraphPad Software, La Jolla, CA). Specific statistical evaluations are indicated in figure legends; significance was concluded when P<0.05.

## Results

### Intranasal dosing volume impacts the efficiency of lung infection in the murine pneumonic plague model

**Fig 1:**
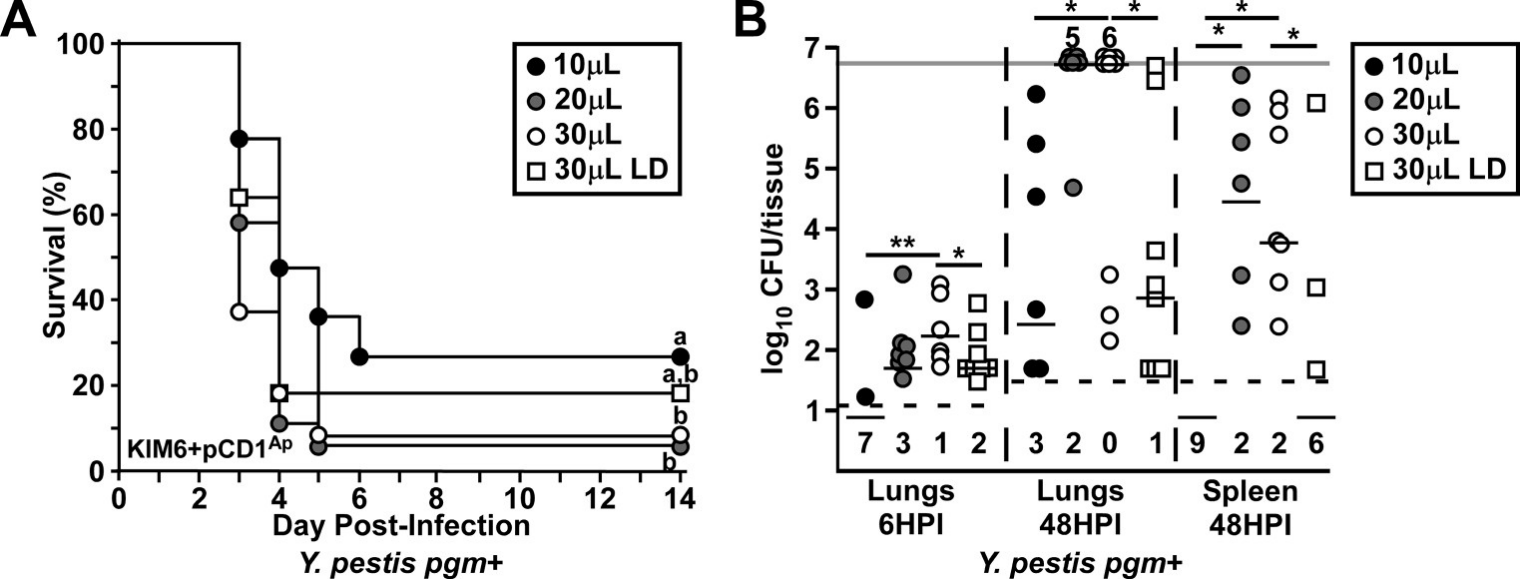
Primary lung infection is affected by volume in the intranasal challenge mouse model. (A-B) Groups of 5–9 C57Bl/6J mice were challenged by intranasal instillation with 2,000 (circles) or 500 (squares) colony forming units (CFU) in 10μL (black), 20μL (grey), or 30μL (white) of *Yersinia pestis* KIM6+pCD1^Ap^, a wild type strain. (A) Survival was monitored for 14 days; n = 16–21 per group, collected in 3 independent trials. Data were combined and analyzed by Mantel-Cox log rank test, survival curves with the same letter are not significantly different. (B) Mice were euthanized at 6 hours post infection (HPI) and CFU in the lungs or at 48 HPI and CFU in the lungs and spleen determined. Dashed line indicates the lower limit of detection, with numbers below that line representing undetected animals for that group; light grey line indicates the upper limit. Data were collected in two independent experiments and each tissue analyzed individually, 2,000 CFU challenge groups by Kruskal-Wallis test with Dunn’s multiple comparisons test and 30μL 500 vs 2,000 CFU groups by Mann Whitney test, *P<0.05, **P<0.01.

Previous work has established that low dose (10μL) intranasal treatments or pathogen challenge results in a high degree of upper respiratory tract infection whereas >30μL result in improved deposition in the lower respiratory tract [22]. To evaluate the impact of volume on intranasal challenge, we compared the response of mice to the same challenge dose of *Y*. *pestis* KIM6+pCD1^Ap^, a wild type strain of the *Mediavalis* biovar, delivered in 10, 20, or 30μL (Fig 1A). At a dose of 2,000 CFU (approximately 6xLD_50_ [15]), there was a small but significant increase in lethality (90% over 5 days) when delivered as 30μL compared to 10μL (70% over 6 days). Similar to 30μL, we observed 90% lethality over 5 days when using 20μL. Reducing the dose by 4-fold and delivering in 30μL (low dose, LD) resulted in similar disease kinetics to 20 and 30μL, with 80% lethality. We measured bacterial titer in the lungs at 6 hours post-infection (HPI) to compare lung deposition of these challenges (Fig 1B). Mice in the high volume groups had deposition of *Y*. *pestis* of at least 10% of the challenge dose in 90% of the 2,000 CFU and 80% of the 500 CFU doses. In striking contrast, the 10μL group had very poor deposition (approximately 1,000-fold lower), and bacteria could not be recovered from the lungs of most of these mice. The 20μL instillation volume led to the greatest variability in deposition amongst challenge conditions and was therefore not significantly different from either 10μL or 30μL challenge. The amount of bacteria recovered at 48 HPI largely correlated with the deposition values, with increased titers in the 30μL group compared to 10μL and 30μL low dose challenges. Bacteria were not recovered in the spleen at 48 HPI in the 10μL group whereas they were commonly observed in the spleens of mice in the 20μL and 30μL groups, suggesting that the infection of secondary tissues was indirectly impacted by dosing volume. Overall, these data suggest that the frequency of primary pneumonic plague may be improved at high volume.

**Fig 2:**
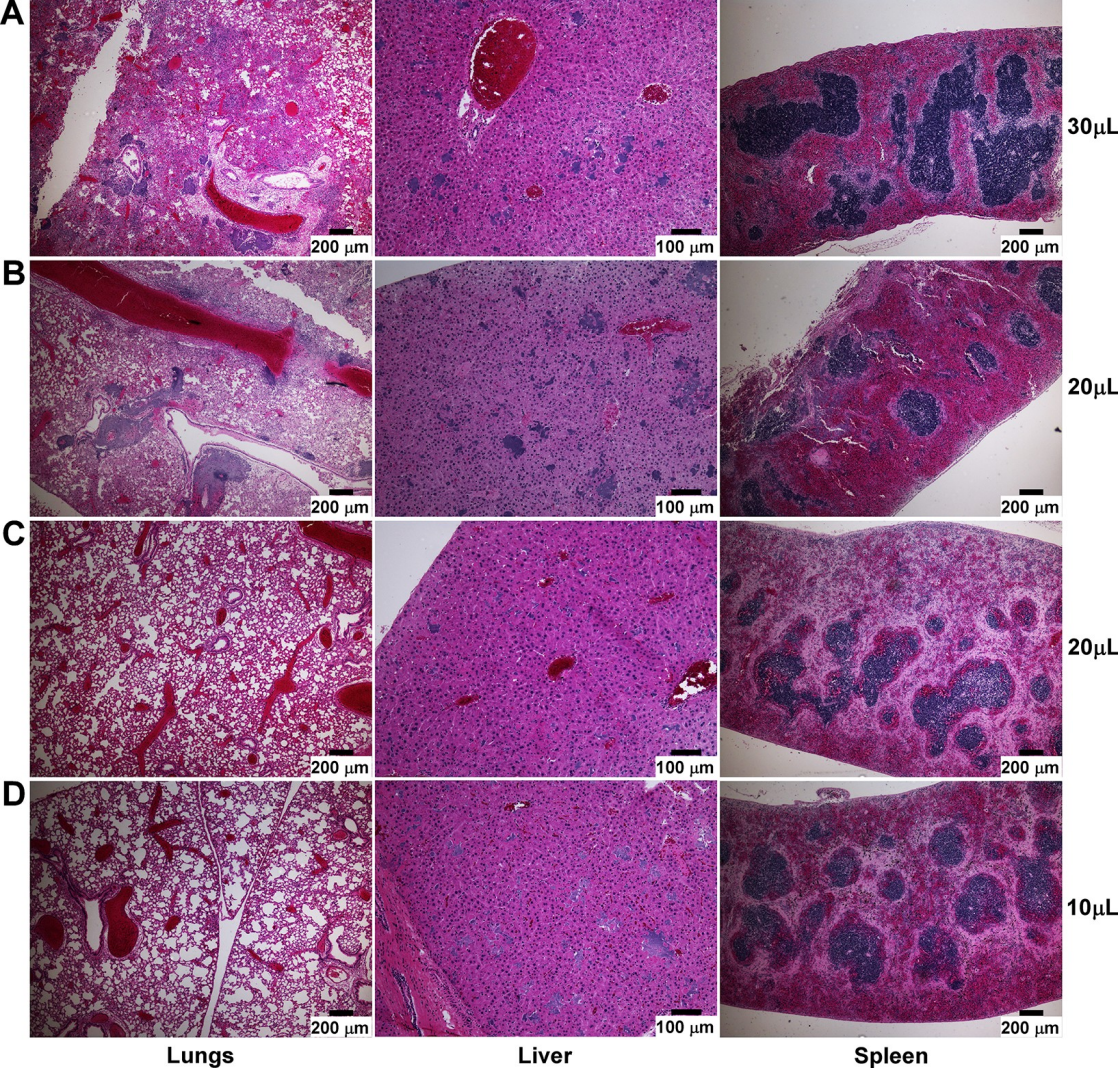
Frequency of septicemic plague in the absence of primary pneumonia increases with decreasing dosing volume. Representative lesions from mice challenged with 2,000 CFU of wild type *Y*. *pestis* in Fig 1 that succumbed to disease on day 4. Formalin fixed tissues collected at necropsy were sectioned and stained with hematoxylin and eosin (H&E). (A) 30μL group; (B-C) 20μL group, high (B) and low (C) severity scores in the lungs; (D) 10μL group. Scale bar represents 100μm (liver) or 200μm (lungs and spleen).

**Fig 3:**
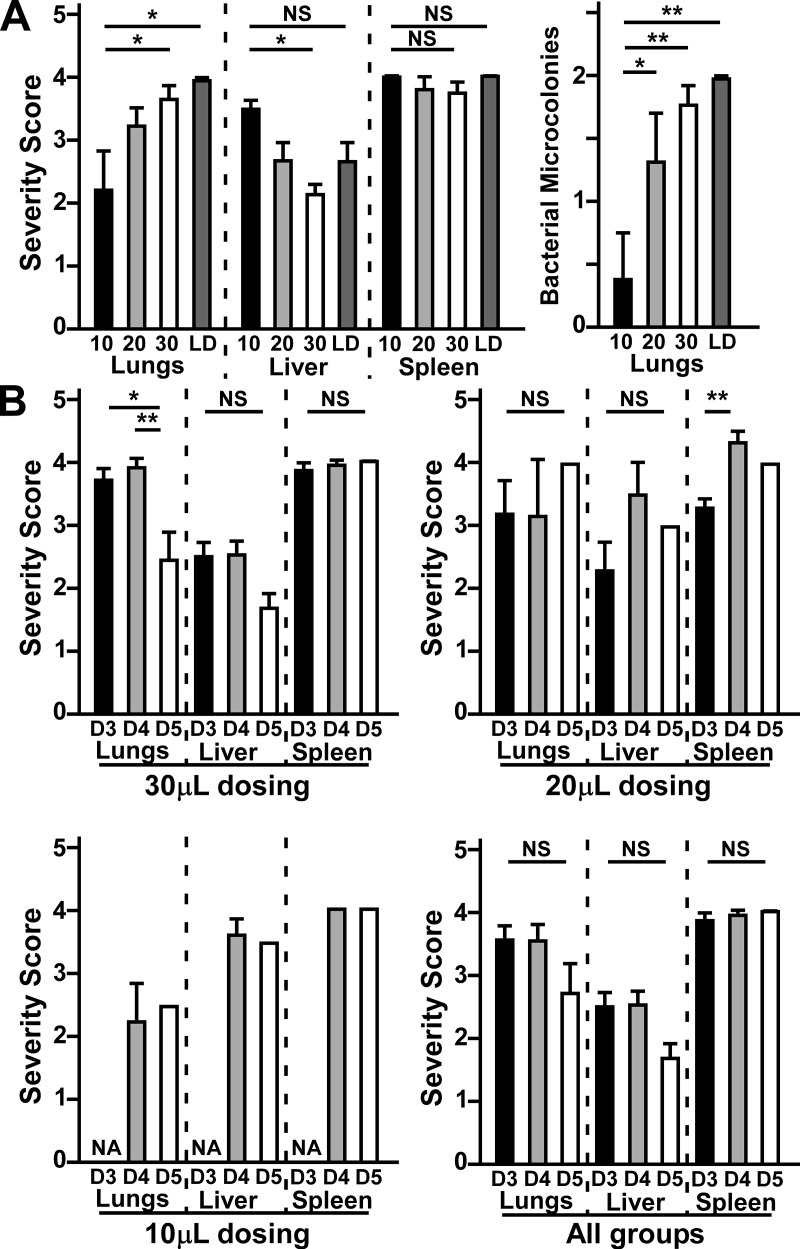
Severity scoring for pathological lesions in mice challenged with wild type *Y*. *pestis* indicates volume and time dependent differences in lung and liver pathology. Tissues collected as described in Fig 2 were scored for severity and frequency of inflammatory and necrotic lesions (0–4.5), as well as the size and frequency of microcolonies (0–2) of *Y*. *pestis* if present. (A) Comparison of lesion severity (left) and bacterial microcolonies (right) of mice challenged with the same dose in 10 (black, n = 5), 20 (grey, n = 9) or 30μL (white, n = 15) or at 4-fold lower dose in 30μL (LD, dark grey, n = 6). (B) Comparison of total lesion severity of mice from 30μL dosing (top left), 20μL dosing (top right), 10μL dosing (bottom left), or combined from all groups (bottom right) that succumbed on day 3 (D3), day 4 (D4) or day 5 (D5). Data shown were collected in 2–3 independent trials and combined for statistical evaluation by one-way ANOVA followed by Tukey’s multiple comparison’s test, *P<0.05, **P<0.01, NS: not significant. Bars represent standard error.

To identify the likely cause of mortality in these animals, we necropsied mice that had succumbed to disease and analyzed lungs, liver and spleen by histopathology. Mice in the 30μL groups, whether they were challenged with 500 or 2,000 CFU *Y*. *pestis*, developed severe lung disease and bacterial microcolonies that were readily observed in all of the mice examined (Fig 2A, 2,000 CFU shown). Bacterial microcolonies could be seen in the liver of these mice, but there was mild pathology in the liver and moderate to severe pathology in the spleens. In the 20μL group, whereas some of the mice developed lung disease similar to 30μL (Fig 2B), there was variability such that some mice in this group had only mild lung disease (Fig 2C). Inflammation and tissue necrosis were similar in the liver and spleen of these mice, regardless of lung pathology. In the 10μL challenge group, mild pathology in the lungs was present and none of the mice examined had developed bronchopneumonia before death, however the livers had moderate to severe inflammatory and necrotic lesions (Fig 2D). Tissues were blind-scored for severity of inflammatory lesions, alveolar necrosis, edema, and hemorrhage. Mice challenged with 10μL had low lesion severity in the lungs and moderate to high lesion severity in the liver, whereas mice challenged with 30μL had significantly higher lesion severity in the lungs with abundant bacteria and lower lesion severity in the liver (Fig 3A). No detectable differences in pathology were noted in the spleens between groups. Overall, the necropsies indicate the highest frequency of primary pneumonic plague in the 30μL groups, with occasional mice with only mild lung disease in the 20μL group.

To determine whether increased disease kinetics correlated with lung disease or secondary infection, we also compared lesion severity in mice that succumbed on days 3, 4 or 5 post-infection (Fig 3B). When comparing only the 30μL groups, we found strong correlation between severity score in the lungs and death on days 3 or 4). Mice that succumbed to infection on day 5, however, showed significantly reduced lesion severity in the lungs. In contrast, comparison of the 20μL groups showed no significant differences in lung pathology over time, nor where there differences in the liver. In the spleens, however, there was a significant increase in pathology between days 3 and 4. There was likewise no changes in pathology between days 4 and 5 in the 10μL groups. Combination of all three increased variability such that the time to death differences in lung and spleen pathology were no longer significant. Overall, the data indicate that 30μL dosing resulted in the most efficient and uniform lung infection that rapidly progressed to bronchopneumonia in addition to secondary septicemic disease, while very little lung bacterial deposition in the 10μL group led to septicemic plague for most if not all affected animals.

**Fig 4:**
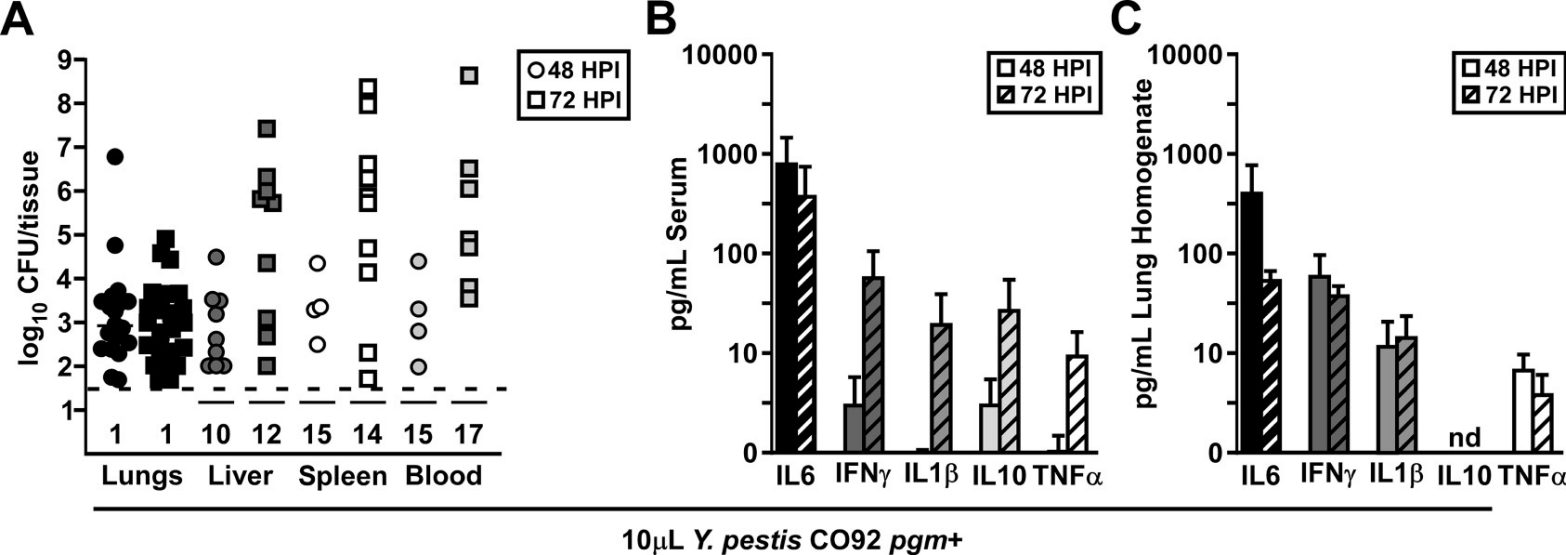
Low volume intranasal challenge with wild type *Y*. *pestis* results in secondary septicemic plague. Groups of 5–6 C57Bl/6J mice were challenged by intranasal infection with 10μL containing 2,000 CFU wild type *Yersinia pestis* CO92. Mice were euthanized at 48 (circles) and 72 (squares) HPI, lungs, liver, spleen, and blood were collected, and (A) bacterial load was quantified, and cytokine concentration in the (B) serum and (C) lungs determined by a multiplex cytokine assay; data shown were collected in 4 independent trials, n = 19 (48 HPI) and 26 (72 HPI).

To verify that mice challenged by intranasal infection with 10μL wild type *Y*. *pestis* developed septicemic plague with little lung involvement, we measured bacterial CFUs in the lungs, liver, spleen, and blood as well as inflammatory cytokines in the serum and lung homogenate after intranasal challenge of mice with 2,000 CFU wild type *Y*. *pestis* CO92, (approximately 5xLD_50_ [29]). To determine changes as disease progressed, we measured CFU and cytokines at 48 and 72 HPI. Consistently, bacterial titer in the liver, spleen and blood appeared higher at 72 HPI than it was at 48 HPI, indicating bacterial growth in these tissues, whereas in the lungs there were no changes in CFU (Fig 4A). Notably, however, about 50% of the mice at both time points had undetectable bacterial titers in the secondary tissues and blood, correlating with the prolonged time to death kinetics of this dosing volume. In contrast, serum cytokines increased over time, with high levels of serum IL6 at both 48 and 72 HPI (Fig 4B). At 72 HPI, sepsis appeared to have progressed, with an increase in serum IFNγ, IL1β, IL10, and TNFα. However, no differences in lung cytokines were apparent between 48 or 72 HPI, indicating pulmonary disease was not progressing in the same manner (Fig 4C). These data further suggest that mice challenged by intranasal infection with 10μL wild type *Y*. *pestis* eventually succumb to secondary sepsis.

### Increased lung deposition of non-pigmented *Y*. *pestis* does not induce primary pneumonic plague

**Fig 5:**
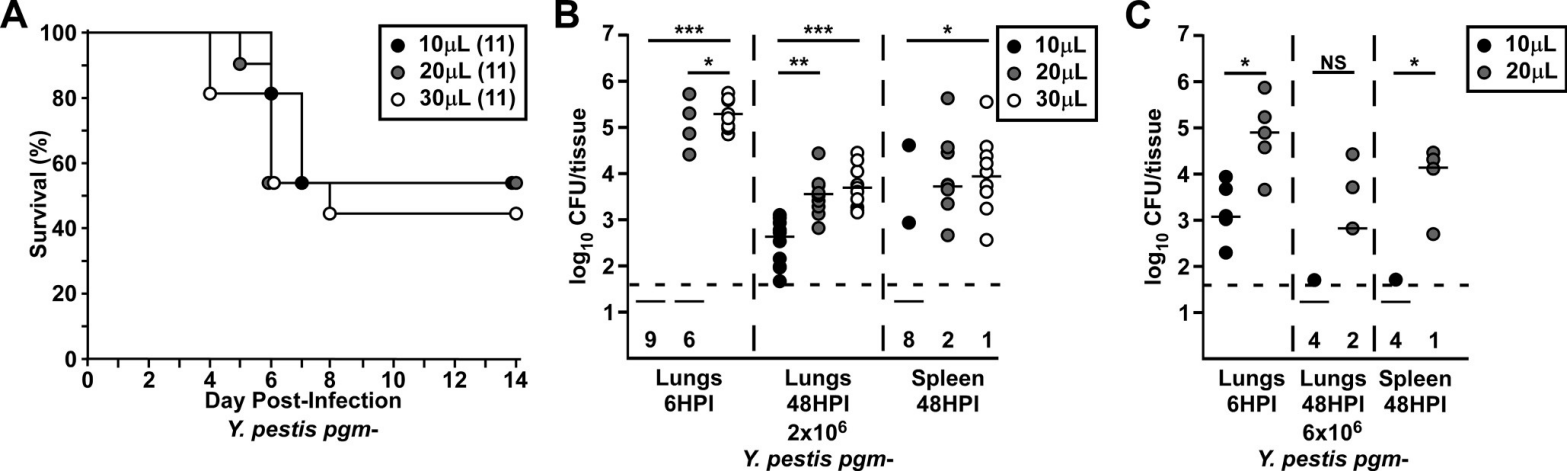
Intranasal challenge with 10 or 20μL *Y*. *pestis pgm*- leads to poor deposition compared to 30μL in the lower respiratory tract but similar survival rates. Groups of C57Bl/6J mice were challenged in parallel with the same inoculation dose of an attenuated *Yersinia pestis pgm*- strain in 10, 20, or 30μL. (A) Mice were challenged with 2x10^6^ CFU and survival was monitored for 14 days. Data from two independent trials were combined and analyzed by Mantel-Cox log-rank test but were not significantly different between groups. (B&C) Animals were euthanized 6 or 48 hours post infection and CFU in the lungs and spleen determined. (B) Data were combined from two independent trials and each tissue analyzed individually by Kruskal-Wallis test with Dunn’s multiple comparison’s test, *P<0.05, **P<0.01, ***P<0.001. (C) Data from one independent trial with each tissue analyzed by Mann Whitney test, *P<0.05, NS: not significant.

Since 30μL wild type *Y*. *pestis* resulted in substantially improved deposition, we asked if there was an impact of volume in the BSL2 model. We repeated the survival, infection and pathological analyses following pulmonary infection with 2x10^6^ CFU of *Y*. *pestis* KIM5-pCD1^Ap^, a *pgm*- strain routinely used as a BSL2 model for plague, delivered in 10, 20, or 30μL. Challenge with 30μL *Y*. *pestis pgm*- resulted in a small, but not significant, increase in lethality compared to 10μL and 20μL (Fig 5A). Bacterial deposition in the lungs showed 1–10% bacterial deposition in 100% of the mice in the 30μL group, whereas all the mice in the 10μL and 60% of the 20μL groups harbored undetectable bacteria in the lungs at 6 HPI, a >5-log difference in titer (Fig 5B). Since approximately 50% of the mice in all the groups develop lethal disease, this result suggests that bacterial deposition in the lungs does not correlate with survival against intranasal challenge with *pgm*- *Y*. *pestis*. At 48 HPI, equivalent titers were recovered from the lungs of mice in the 20 and 30μL groups, each of which was significantly higher than the 10μL group. Fewer mice in the 10μL group had bacteria in the secondary tissues, whereas this occurred with similar frequency in the 20 and 30μL groups. Only a 3-fold increase in challenge dose improved recovery of bacteria in the lungs of mice in the 10 and 20μL groups, such that bacteria were recovered in the lungs of all the mice at 6 HPI (Fig 5C). By 48 HPI, there was a decrease in pulmonary bacteria in both groups. Thus, the *pgm* mutant does not cause pneumonia even when deposition is high. Overall, these results suggest that large differences in lower respiratory tract deposition do not substantially alter the outcome of infection of *pgm*- *Y*. *pestis*.

**Fig 6:**
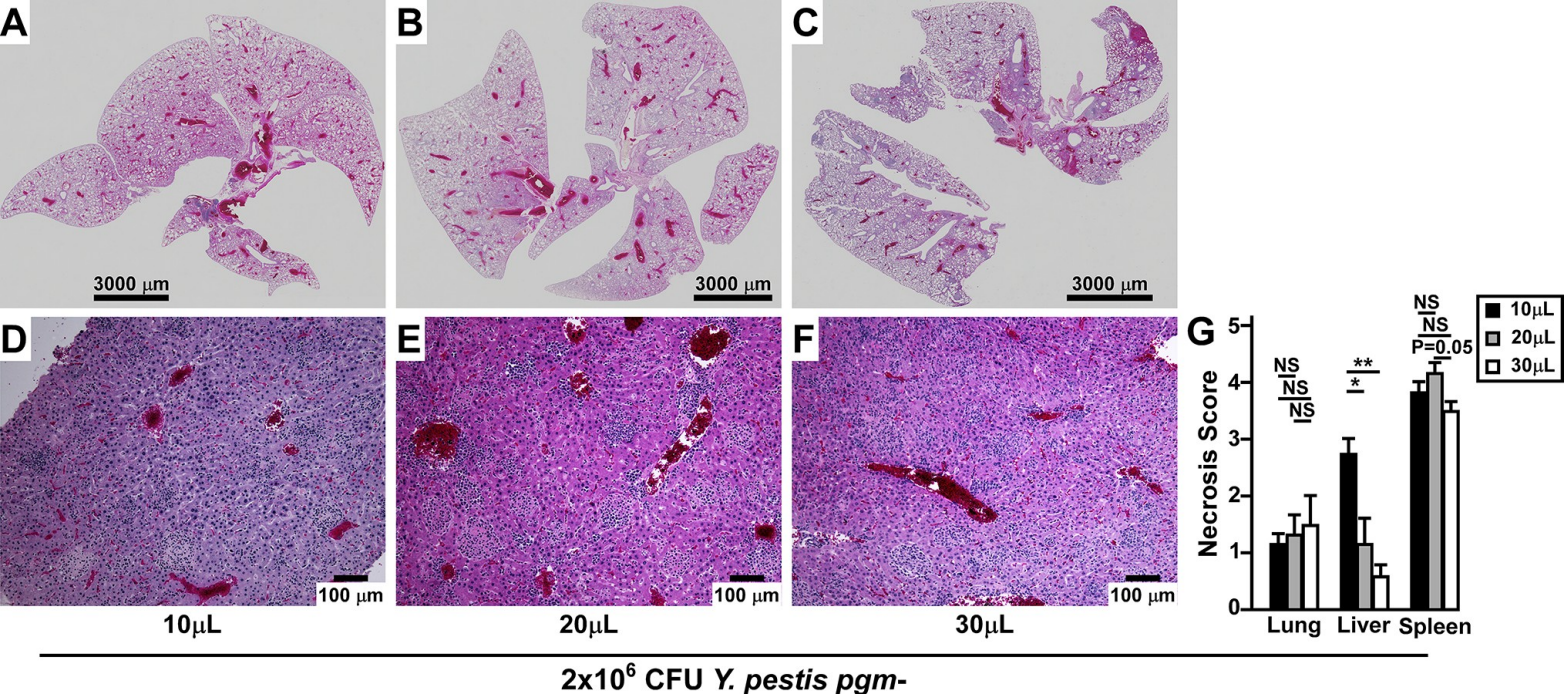
Increased liver necrosis but no lung pathology during low volume intranasal challenge with non-pigmented *Y*. *pestis*. Groups of mice challenged in Fig 4 were analyzed post-mortem histopathology of formalin fixed tissues. Whole lungs (A-C) and representative liver sections (D-F) from 10μL (A&D), 20μL (B&E) and 30μL (C&F) challenges, scale bar represents 3,000μm (A-C) or 100μm (E-F). (G) Severity scoring on total lesion severity in the lungs or necrosis severity in the liver and spleen. Data shown were pooled from two independent trials; n = 3 per group, 10μL and 20μL; n = 5 per group, 30μL; error bars indicate standard error. Data were evaluated by one-way ANOVA followed by Tukey’s multiple comparisons test, *P<0.05, **P<0.01, NS: not significant.

Mice that succumbed to disease were analyzed post-mortem by histopathology to identify likely cause of death. Consistent with previous results, we did not find any mice in any group with severe lung disease, even when whole lung sections were examined (Fig 6A–6C). Mice in the 30μL group occasionally harbored moderate inflammatory lesions, but bacterial microcolonies were not visible, and there were no significant differences in severity scores of lung pathology between dosing groups (Fig 6G). Similarly, mice in all groups had many inflammatory lesions in the liver, yet increased hepatocyte necrosis was observed in the 10μL group compared to the 20μL or 30μL groups (Fig 6D–6F). Mean severity scores from each tissue were compared between dosing groups (Fig 6G). While overall lesion severity was similar in the liver, significantly more necrosis was present in the 10μL group. As hepatocyte necrosis occurs during sepsis, this observation suggests that increased retention of *Y*. *pestis* in the upper respiratory tract may lead to the earlier development of septicemic plague.

**Fig 7:**
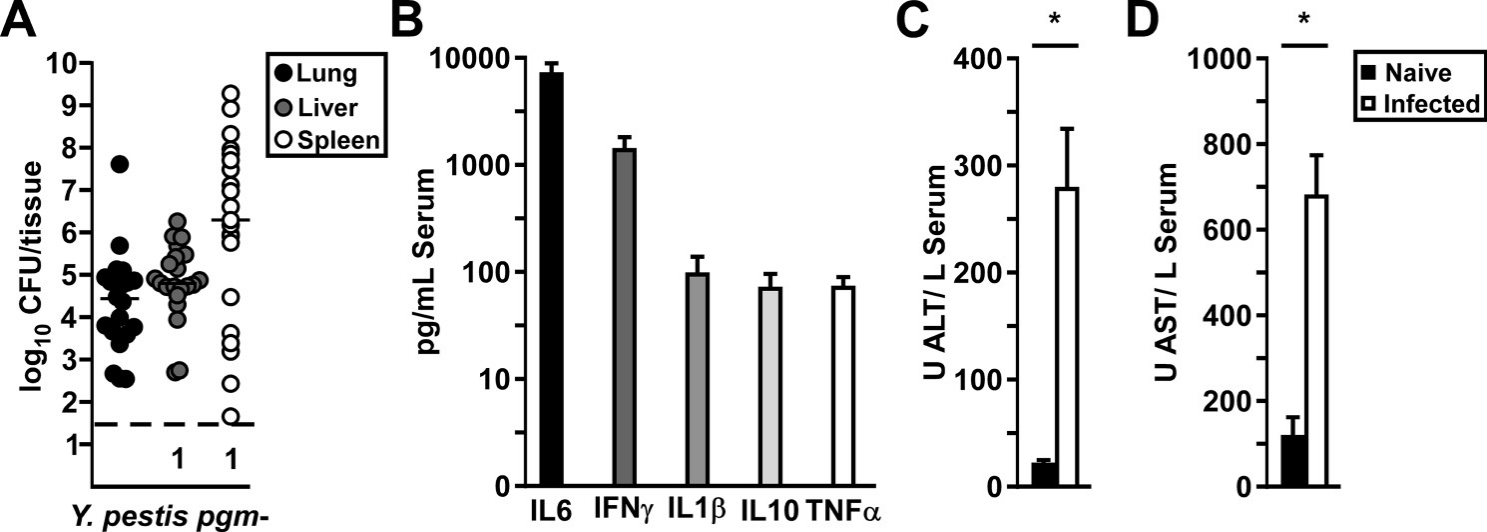
Low volume intranasal challenge causes variable infection but high frequency sepsis and liver damage. Groups of 5 wild type C57Bl/6 mice were challenged by intranasal infection of *Y*. *pestis* KIM5-pCD1^Ap^ in 10μL volume. On day 5 post-infection, mice were euthanized, blood and tissues collected and processed for (A) bacterial titer; (B) serum cytokines by a multiplex cytokine assay; (C-D) serum liver function enzyme panel. Data shown were collected in 2–4 independent trials, n = 20 (A-B) or n = 10 (C-D); bars in (A) indicate median, in (B-D), bars indicate standard error. Pooled data (C-D) were analyzed by Kruskal Wallis, *P<0.05.

To confirm this, we measured bacterial titers, serum cytokines and liver function enzymes during the disease phase in mice challenged by intranasal infection with 10μL of 2x10^6^ CFU *Y*. *pestis* KIM5-pCD1^Ap^. On day 5 post-infection, bacterial titers in the liver were moderate, whereas in the spleen there was large variation in titer of over 7-logs (Fig 7A). Serum cytokines, including IL6, IFNγ, IL1β, IL10, and TNFα were high suggesting the onset of sepsis in most of these mice even though there was a high degree of variation of bacterial load (Fig 7B). Liver function enzymes, including AST and ALT which are indicators of tissue injury, were significantly higher than naïve mice (Fig 7C and 7D) [30]. Overall the data are consistent with 10μL challenge volumes leading to the development of lethal septicemic plague, independent of the primary lung infection.

## Discussion

In this work, we identified a significant impact of dosing volume on the development of pneumonic or septicemic plague as the likely cause of death following intranasal challenge of mice with wild type *Y*. *pestis*. These data have important implications on plague research and the early testing of candidates for new vaccines, therapeutics and diagnostics. Retention in the upper respiratory tract that results from low volume intranasal instillation leads to a high frequency of secondary septicemic plague, with multi-organ failure as evidenced by severe hepatocyte necrosis and elevation of cytokines and liver enzymes in the serum in mice as they succumb to infection. Presumably this occurs via the nasal sinuses when a high degree of bacteria reside in the nasal passages. In contrast, high volume administration results in reproducibly uniform bacterial deposition in the lower respiratory tract and a high frequency of primary pneumonic plague, with bacterial microcolonies but few inflammatory foci in the secondary tissues of moribund mice. Similar pathology in lungs, liver and spleen was reported in moribund mice that had been aerosol challenged indicating high volume intranasal challenge is a reasonable substitute for aerosol [18]. With isoflurane anesthesia, we routinely observed 5–10% deposition using 30μL dosing volume, and even very low doses resulted in high fidelity lower respiratory tract deposition and disease. Furthermore, we found that 20μL dosing volume, a method commonly used in the field, was more similar to 30μL than it was to 10μL, but resulted in more variability, mixed results, and reduced the power of the study.

Even with efficient deposition to the lungs, the *pgm* mutant was unable to cause bronchopneumonia. We were surprised to find that even 2x10^6^ CFU of *Y*. *pestis pgm*- was only infrequently recovered from the lungs at 6 HPI in the low volume challenge, whereas in the high volume challenge, we recovered high amounts of bacteria from the lower respiratory tract. These differences did not correlate with lethality consistent with sepsis as a primary cause of death with little involvement from the primary infected tissue. Furthermore, *pgm*- *Y*. *pestis* were rapidly cleared from the lungs, suggesting a major role for the *pgm* locus in evasion of immune clearance from the lungs. Previous work established that *pgm*-encoded yersiniabactin was necessary for bacterial growth in the lungs, and that additional virulence functions were present in the *pgm* locus [15]. The data shown here support the hypothesis that these additional virulence functions may allow for immune evasion in the lungs and perhaps other infected tissues.

To date, there is little known about septicemic plague caused by wild type *Y*. *pestis*, probably due to the high biosafety risk of intravenous infection of mice. The data shown here indicate low volume intranasal challenge of wild type *Y*. *pestis* presents an alternative to intravenous challenge for modeling septicemic plague and could be considered for future work on pathogenesis, vaccine and therapeutic development as a method for understanding how *Y*. *pestis* induces lethality by septicemia.
